# Supplementary material for: Higher SARS-CoV-2 Spike Binding Antibody Levels and Neutralization Capacity 6 Months after Heterologous Vaccination with AZD1222 and BNT162b2
Source: Vaccines (Basel). 2022 Feb 17;10(2):322. doi: 10.3390/vaccines10020322 (PMC8880180; doi:10.3390/vaccines10020322)
Supplement: Supplementary file 1 [file vaccines-10-00322-s001.zip › vaccines-1574208-supplementary.pdf]

**Table S1. anti-spike binding antibody units (BAU/mL)**

| | AZD1222 / AZD1222 | AZD1222 / BNT162b2 | BNT162b2 / BNT162b2 | all (n=190) |
|---|---|---|---|---|
| *sex differences* | | | | |
| male: median (min-max) | 562.3 (98.8 – 2140) | 2029 (1092 – 4273) | 824.6 (137.6 – 2206) | 830 (98.8 – 4273) |
| female: median (min-max) | 403.8 (35.6 – 3023) | 2234.5 (199.2 – 24762) | 661.9 (178.9 – 2580) | 968.5 (35.6 – 24762) |
| p (Mann-Whitney U test) | 0.1266 | 0.8492 | 0.5875 | 0.1959 |
| *correlation with age* | | | | |
| r (spearman rank) | r = -0.1220 95% | r = -0.07360 | **r = -0.3989** | **r = -0.2338** |
| 95 % CI | -0.3693 to 0.1415 | -0.3224 to 0.1847 | **-0.5894 to -0.1665** | **-0.3677 to -0.09033** |
| p-value | 0.3490 | 0.5665 | **0.0009** | **0.0012** |
| *correlation with % neutralization capacity* | | | | |
| r (spearman rank) | **r = 0.4718** | **r = 0.6701** | **r = 0.8032** | **r = 0.8033** |
| 95 % CI | **0.2425 to 0.6513** | **0.5007 to 0.7900** | **0.6927 to 0.8768** | **0.7444 to 0.8498** |
| p-value | **0.0001** | **< 0.0001** | **< 0.0001** | **< 0.0001** |
| *correlation with IFNγ-producing spots* | | | | |
| r (spearman rank) | r = -0.09428 | r = 0.1767 | r = 0.1738 | r = 0.1125 |

| | | | | |
|---|---|---|---|---|
| 95 % CI | -0.3449 to 0.1688 | -0.08177 to 0.4130 | -0.07860 to 0.4052 | -0.03459 to 0.2549 |
| p-value | 0.4698 | 0.1658 | 0.1629 | 0.1222 |

**Table S2. anti-RBD (Wuhan-Hu-1) neutralizing capacity (%)**

| | AZD1222 / AZD1222 | AZD1222 / BNT162b2 | BNT162b2 / BNT162b2 | all (n=190) |
|---|---|---|---|---|
| *sex differences* | | | | |
| male: median (min-max) | 71 (33-95) | 93 (91 – 96) | 87 (38 – 93) | 85 (33 – 96) |
| female: median (min-max) | 51 (16 – 88) | 94 (60 – 96) | 87 (49 – 94) | 87 (16 – 96) |
| p (Mann-Whitney U test) | 0.0558 | 0.4490 | 0.9122 | 0.4130 |
| *correlation with age* | | | | |
| r (spearman rank) | r = -0.1259 | -0.05884 | **r = -0.3307** | **r = -0.1870** |
| 95 % CI | -0.3727 to 0.1376 | -0.3090 to 0.1990 | **-0.5355 to -0.08903** | **-0.3246 to -0.04159** |
| p-value | 0.3337 | 0.6469 | **0.0067** | **0.0098** |
| *correlation with IFNγ-producing spots* | | | | |
| r (spearman rank) | r = 0.1137 | r = 0.008657 | r = 0.1316 | r = 0.1216 |

| | | | | |
|---|---|---|---|---|
| 95 % CI | -0.1497 to 0.3620 | -0.2467 to 0.2629 | -0.1213 to 0.3685 | -0.02538 to 0.2634 |
| p-value | 0.3831 | 0.9463 | 0.2923 | 0.0947 |

**Table S3. anti-RBD (Delta B.1.617.2 variant) neutralizing capacity (%)**

| | AZD1222 / AZD1222 | AZD1222 / BNT162b2 | BNT162b2 / BNT162b2 | all (n=190) |
|---|---|---|---|---|
| *sex differences* | | | | |
| male: median (min-max) | **69 (37-93)** | 93 (85 – 96) | 88 (44 – 96) | 85 (37 – 96) |
| female: median (min-max) | **60.8 (18-87)** | 94 (64 – 96) | 85 (56 – 96) | 85 (18 – 96) |
| p (Mann-Whitney U test) | **0.038** | 0.9207 | 0.7359 | 0.5556 |
| *correlation with age* | | | | |
| r (spearman rank) | r = -0.08332 | -0.02527 | **r = -0.2742** | **r = -0.1584** |
| 95 % CI | -0.3351 to 0.1795 | -0.2783 to 0.2310 | **-0.4897 to -0.2712** | **-0.2980 to -0.01217** |
| p-value | 0.5232 | 0.8441 | **0.0259** | **0.0290** |
| *correlation with IFN-γ-producing spots* | | | | |

| | | | | |
|---|---|---|---|---|
| r (spearman rank) | r = 0.004285 | r = 0.09498 | r = 0.2242 | r = 0.1238 |
| 95 % CI | -0.2550 to 0.2630 | -0.1638 to 0.3415 | -0.0262 to 0.4482 | -0.02318 to 0.2655 |
| p-value | 0.9739 | 0.4590 | 0.0703 | 0.0888 |

**Table S4. IFN⚜-producing spots**

| | AZD1222 / AZD1222 | AZD1222 / BNT162b2 | BNT162b2 / BNT162b2 | all (n=190) |
|---|---|---|---|---|
| *sex differences* | | | | |
| male: median (min-max) | 74 (0 – 102) | 75 (42 – 120) | 54 (6 – 87) | 67 (0 – 120) |
| female: median (min-max) | 63 (0 – 106 | 72.5 (5 – 123) | 58 (6 – 115) | 67 (0 – 123) |
| p (Mann-Whitney U test) | 0.6391 | 0,5321 | 0.2378 | 0.8676 |
| *correlation with age* | | | | |
| r (spearman rank) | r = 0.03235 | r = 0.02503 | r = -0.2037 | r = -0.06716 |

| | | | | |
|---|---|---|---|---|
| 95 % CI | -0.2286 to 0.2889 | -0.2313 to 0.2781 | -0.4308 to 0.04762 | -0.2116 to 0.08017 |
| p-value | 0.8045 | 0.8456 | 0.1008 | 0.3572 |
